# Effects of enteral insulin on enteral diet tolerance in premature infants: systematic review

**DOI:** 10.1590/1984-0462/2025/43/2024115

**Published:** 2024-12-20

**Authors:** Letícia Baciuk-Souza, Laura Ferrer Del Pra, Luca Schiliró Tristão, Mayco José Reinaldi Serra, Vera Esteves Vagnozzi Rullo

**Affiliations:** a Fundação Lusíada, Faculdade de Ciências Médicas de Santos, Santos, SP, Brazil.

**Keywords:** Preterm neonate, Very low birth weight newborn, Insulin, Neonato pré-termo, Recém-nascido de muito baixo peso, Insulina

## Abstract

**Objective:**

The primary objective of this systematic review was to evaluate the effect of enteral insulin supplementation provided to premature newborns on the time to achieve full enteral feeding. Secondary objectives included evaluating the effects on weight gain, the occurrence of adverse events, and mortality.

**Data source:**

A systematic review of randomized clinical trials was conducted using the databases PubMed, Scientific Electronic Library Online (SciELO), clinicaltrials.gov, Embase, and Latin American and Caribbean Health Sciences Literature (Lilacs). The review was registered in PROSPERO under CRD42024523021. Premature newborns receiving enteral and parenteral nutrition who were given either breast milk or formula milk supplemented with enteral insulin as intervention, compared with placebo, were included. After applying the eligibility criteria, two articles were selected for this study.

**Data synthesis:**

The two studies included in this review identified a reduced time to achieve full enteral feeding. Regarding secondary outcomes, there was a reduction in the number of days receiving parenteral nutrition and a reduction in adverse events and mortality. However, there was no agreement among the studies concerning weight gain.

**Conclusions:**

Enteral insulin appears to be an effective treatment for reducing the time to achieve full enteral feeding. However, more studies are necessary to recommend its use in clinical practice.

## RESUMO

**Objetivo:**

O objetivo primário desta revisão sistemática foi avaliar o efeito da suplementação com insulina enteral fornecida a recém-nascidos prematuros sobre o tempo para obtenção de dieta enteral plena. Os objetivos secundários incluíram avaliar os efeitos sobre o ganho de peso, a ocorrência de eventos adversos e a mortalidade.

**Fontes de dados:**

Uma revisão sistemática de ensaios clínicos randomizados foi realizada nas bases de dados PubMed, *Scientific Electronic Library Online* (SciELO), clinicaltrials.gov, Embase e *Latin American and Caribbean Health Sciences Literature* (Lilacs). A revisão foi registrada na PROSPERO sob o CRD42024523021. Foram incluídos recém-nascidos prematuros em uso de dieta enteral e parenteral que receberam como intervenção leite materno ou fórmula láctea suplementada com insulina enteral, comparados com placebo. Após aplicação dos critérios de elegibilidade, dois artigos foram selecionados para o presente estudo.

**Síntese dos dados:**

Os dois estudos incluídos nesta revisão identificaram redução do tempo para obtenção de dieta enteral plena. Com relação aos desfechos secundários, houve redução do número de dias recebendo nutrição parenteral e redução de eventos adversos e mortalidade. Porém, com relação ao ganho de peso não houve concordância entre os estudos.

**Conclusões:**

A insulina enteral parece ser um tratamento eficaz em reduzir o tempo para obtenção de dieta enteral plena. Porém, mais estudos são necessários para recomendar seu uso na prática clínica.

## INTRODUCTION

Establishing adequate nutrition is a challenge for many premature newborns.^1^ The Pan American Health Organization (PAHO) recommends that breast milk be the first and only food for newborns up to six months of age, including for premature infants.^2^ This recommendation is because breast milk provides benefits beyond adequate nutrition, being rich in bioactive factors such as hormones, immunoglobulins, and probiotics, among others, with various biological functions.^3^

Complications related to prematurity or low birth weight appear to be influenced by dietary factors, such as the use of formula or donated human milk, compared to breast milk. Studies indicate that concentrations of bioactive factors (e.g., peptide hormone insulin, lactoferrin, and immunoglobulins) are higher in colostrum, decrease during lactation, are absent in formula, and are reduced in donated human milk.^3,4^

Unfortunately, prematurity is associated with difficulty in enteral feeding or food intolerance, due to the immaturity of the gastrointestinal tract (GIT), often requiring fluids and parenteral nutrition.^5,6^ It is known that prolonged exposure of these patients to parenteral nutrition is associated with a higher risk of complications such as sepsis, necrotizing enterocolitis, and retinopathy of prematurity, among others,^7^ in addition to a higher risk of poor growth and developmental delay.^8^

In the long term, health repercussions such as a higher risk of obesity, systemic arterial hypertension, and type 2 diabetes mellitus can also be observed.^5,8^

Considering the deleterious effects of prolonged parenteral nutrition, the search for a method that reduces the need for this nutritional method or reduces the exposure time has proven to be extremely relevant.^9^

Due to the presence of insulin in the amniotic fluid, colostrum, and mature breast milk, it has been speculated that insulin might have a biological effect of accelerating or encouraging enterocytological maturation throughout the GIT.^10,11^ Preclinical trials have confirmed that insulin not only plays a role in stimulating enterocyte proliferation but also in reducing apoptosis and increasing enzyme activity in the intestinal microvilli.^7,12,13^

In this context, this systematic review aimed to evaluate the effect of enteral insulin supplementation provided to preterm infants on the time to achieve full enteral feeding (FEF). As secondary objectives, effects on weight gain, the occurrence of adverse events, and mortality were also assessed.

## METHOD

This study is a systematic review of the literature to evaluate whether the use of enteral insulin in premature newborns improves gastrointestinal tolerance, reducing the need for parenteral nutrition.

This review followed the Preferred Reporting Items for Systematic Reviews and Meta-Analyses (PRISMA) guidelines, that helps researchers improve the preparation of systematic reviews, and is registered in PROSPERO under CRD42024523021.^14,15^

Our review started from the following question: What is the effect of enteral insulin on tolerance to enteral feeding?

P: Newborns less than 37 weeks of gestational age.

I: Feeding with breast milk or formula supplemented with enteral insulin.

C: Feeding with breast milk or formula without enteral insulin supplementation.

O: Time to reach FEF, days receiving parenteral nutrition, weight gain in grams, adverse events, and mortality.

Our primary outcome was the time to reach FEF, considering 150 mL/kg/day or more, for three consecutive days. As secondary outcomes, the following were evaluated: days receiving parenteral nutrition, weight gain in grams, adverse events, and mortality.

A systematic literature review was conducted on the databases Medline (PubMed), Scientific Electronic Library Online (SciELO), clinicaltrials.gov, Embase, and Latin American and Caribbean Health Sciences Literature (Lilacs/BVS). The search strategy was: (Premature Infant OR Preterm Infant OR Neonatal Prematurity OR Low-Birth-Weight Infant OR Low Birth Weight Infant OR Low Birth Weight) AND insulin.

The articles were selected by two researchers according to the following inclusion criteria present in the title and/or abstract: (I) Premature newborns; (II) Combined enteral and parenteral diets; and (III) Randomized clinical trials. The exclusion criteria were: (I) Children with major congenital malformations; (II) Suspected necrotizing enterocolitis; (III) Suspected neonatal sepsis; (IV) Use of parenteral insulin; and (V) Presence of persistent hyperinsulinemic hypoglycemia. There were no restrictions regarding the period or language of publication. In the consensus meetings with the other authors, there were no discrepancies.

The risk of bias of the randomized clinical trials was assessed through the Revised Cochrane Risk-of-Bias Tool for Randomized Trials (RoB2) and is summarized in Table 1.^16^

**Table 1:** Risk of bias of randomized clinical trials using the tool Revised Cochrane Risk-of-Bias Tool for Randomized Trials (RoB2)^16^.

Author	Domain 1: Randomization Process	Domain 2: Intended Intervention	Domain 3: Incomplete Data	Domain 4: Outcome Measurement	Domain 5: Outcome Reporting	Overall analysis
Mank et al.^7^	Low	Low	Low	Low	Some concerns	Some concerns
Shehadeh et al.^18^	Low	Low	Low	Low	Low	Low

## RESULTS

**Figure 1:**
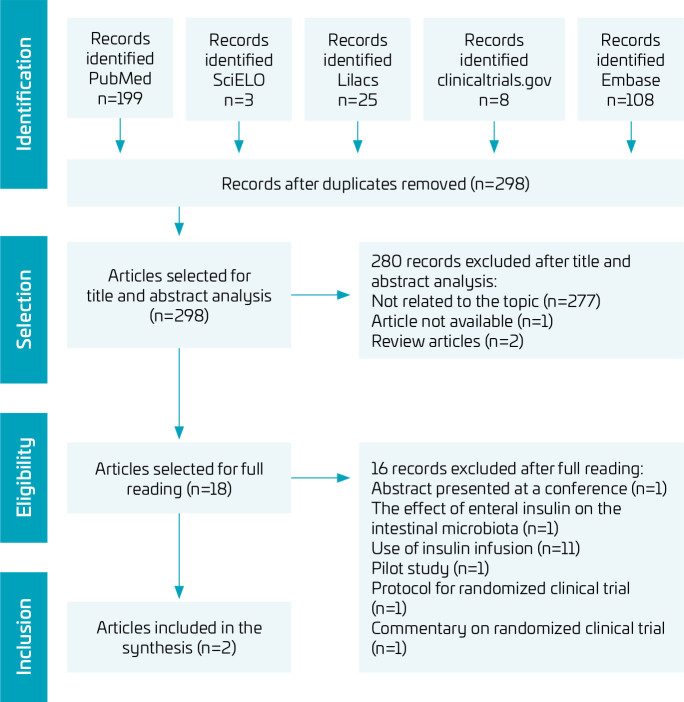
Flowchart based on the PRISMA model with the results of the article selection.

Source: Prepared by the author.

The search conducted up to April 2024 retrieved 298 articles after duplicates removal. Of these, 18 were selected for full-text reading. According to eligibility criteria, two articles were included in this systematic review (Figure 1), and their main characteristics are presented in Table 2.

**Table 2:** Synthesis of the articles included in the review.

Author/Country	Main inclusion criterion	Randomized participants	Interventions	Primary outcome	Results	p-value
Mank et al.^7^ Netherlands	Premature babies with 26–32 weeks of gestation, birth weight ≥500 grams. GA: low dose of rh insulin (400 µIU/mL of milk) GB: high dose of rh insulin (2000 µIU/mL of milk) GC: placebo	GA: 110 (108 included) GB: 95 (88 included) GC: 98 (97 included)	Rh insulin (powder) developed for enteral administration (ELGAN Pharma). GA: sachet of 0.04 IU/g of rh insulin. GB: sachet of 0.2 IU/g of rh insulin. GC: manufactured identically but with no inclusion of rh insulin.	Time to achieve FEF, defined as an enteral intake of at least 150 mL/kg per day for three consecutive days.	Comparing GA and GB with GC; the median time (IQR) to achieve FEF was reduced: GA in 94 infants (10.0 [7.0–21.8] days) GB in 82 infants (10.0 [6.0–15.0] days) GC in 85 babies who received placebo (14.0 [8.0–28.0] days). Difference in average time (95%CI) for FEF: GA=4.0 (1.0–8.0) days GB=4.0 (1.0–7.0) days. Primary outcome remained significant for both doses of rh insulin after adjustment for multiple tests.	p<0.050 p=0.030 p=0.001 p<0.050
Shehadeh et al.^18^ Israel	Premature babies with 26–33 weeks of gestation, birth weight ≥750 grams. GA: received 400 µU/ml insulin mixed with enteral feeding. GC: received placebo added to their formula.	GA: 17 (16 included) GC: 16 (15 included)	Infants were randomized 1:1 to receive insulin (GA) or placebo (GC). The investigated insulin (NTRA, Nutrinia Ltd., Israel) was a dry powder composed of human insulin (rDNA origin) and microencapsulated in a maltodextrin matrix, at an insulin dose of 400 µU/ml of enteral feeding.	Number of days required to achieve full enteral feeding, defined as enteral consumption of 150 ml/kg/day of infant formula for preterm infants.	Average time for complete enteral feeding: GA: 6.37 days (95%CI 4.59–8.15) GC: 8.00 days (95%CI 6.20–9.80) Difference between the groups: 1.63 days (95%CI 0.29–2.97)	p=0.023

GA: Group A; GB: Group B; GC: Control Group; FEF: full enteral feeding; IQR: interquartile range.

Source: Developed by the authors.

In the study by Mank et al., 303 premature newborns with 26.0 to 32.0 weeks of gestation and a birth weight of 500g or more were included. The infants were randomized into three groups: Group A (GA) comprised 110 infants (108 included) who received a low dose of recombinant human (rh) insulin (400 μIU/mL of milk); Group B (GB) consisted of 95 infants (88 included) who received a high dose of rh insulin (2000 μIU/mL of milk); and Group C (GC) had 98 infants (97 included) who were given a placebo dose, identically manufactured but without rh insulin. As the data did not follow a normal distribution, the 95%CI of the median difference was computed using bootstrapping. The chi-square (χ^2^) test was employed for categorical variables and a gatekeeping procedure was conducted to adjust for multiple testing. The median age for GA was 29.1 (28.1–30.4) weeks, for GB was 29.0 (27.7–30.5) weeks, and for GC was 28.8 (27.6–30.4) weeks. The birth weight was 1200 g (976–1425), 1250 g (1020–1445), and 1208 g (1021–1430), respectively.^17^

The insulin doses were prepared through a rh insulin powder formulation developed for enteral administration (ELGAN Pharma). The product was packaged in sachets containing 0.5 g each. Next, 200 sachets with identical packaging numbers were packed in sealed boxes. For each participating infant, a standard solution was prepared daily by the researcher, doctor, or neonatal nurse, using one sachet per 1.8 mL of diluent (breast milk, donated human milk, or preterm formula). Subsequently, a daily dosage of 0.04 mL of solution per milliliter of enteral feeding was prescribed for all participating infants to reach the target concentration of rh insulin (400 μIU/mL of milk in the low-dose group; 2000 μIU/mL of milk in the high-dose group; and placebo).

The primary outcome evaluated was the time to achieve FEF, which was reached by 86% of the 303 included infants. Comparing GA and GB with GC, the median time to achieve FEF was reduced: GA in 94 infants (10.0 [7.0–21.8] days; p=0.030); GB in 82 infants (10.0 [6.0–15.0] days; p=0.001); and GC in 85 infants who received placebo (14.0 [8.0–28.0] days). The difference in mean time (95%CI) for FEF was GA 4.0 (1.0–8.0) days and GB 4.0 (1.0–7.0) days. The primary outcome remained significant for both dosages of rh insulin after adjustment for multiple testing (p≤0.050).

The safety outcomes included serious adverse events (SAEs), presented through descriptive statistics. One or more SAEs were present in 15% of infants in GA, 13% in GB, and 20% in GC. Hypoglycemia events manifested in 2% of infants in GA, 3% in GB, and 3% in GC. Necrotizing enterocolitis (Bell stage 2 or 3) occurred in 6% of infants in GA, 5% in GB, and 10% in GC. None of the infants developed serum antibodies against insulin. Regarding mortality, there was no difference between the intervention and placebo groups, with five deaths in the low-dose group, one in the high-dose group, and four in the placebo group.

In the study by Shehadeh et al., 33 premature infants with 26.0 to 33.0 weeks of gestation and birth weight of 750 g or more were included. Newborns up to 7 days of age considered stable enough to tolerate oral feeding were included, restricted to infants fed with formula. The infants were randomized into two groups; Group A (GA) comprised 17 infants (16 included) who received a dose of 400 μU/mL of human insulin added to the infant formula and Group C (GC) consisted of 16 infants (15 included) who received a placebo dose without added rh insulin. The mean age for GA was 30.9 weeks, standard deviation (±)1.5 and for group GC was 30.6±2.1 weeks. The birth weight for GA was 1470.7±299.7 g and for group GC was 1446.8±364.8 g.^18^

The insulin formulation investigated in Shehadeh et al.’s study (NTRA, Nutrinia Ltd., Israel) was a dry powder composed of human insulin (rDNA origin), microencapsulated in a maltodextrin matrix. The insulin dose of 400 μU/mL of enteral feeding was based on the insulin level to which a fetus would be exposed in utero through amniotic fluid. Infant formula was used as diluent to administer 400 μU/mL rh insulin of enteral feed or a placebo via enteral consumption of NTRA.

In the primary outcome, the mean time to FEF, the difference between the groups was 1.63 days (95%CI 0.29–2.97), with GA at 6.37 days (95%CI 4.59–8.15) and GC at 8.00 days (95%CI 6.20–9.80); p=0.023.

For the secondary outcomes, the time to achieve a gastric residual of less than 2.0 mL/kg was 1.67 days for GA (95%CI -1.42–4.76) and 5.09 days for GC (95%CI 1.92–8.26); the difference between the treatment groups was 3.42 days (95%CI 0.12–6.96); p=0.056. The mean weight gain for GA was 768.9 g, and for GC was 643.6 g, representing a 19% weight increase among the infants in the enteral rh insulin group compared to the placebo group.

The safety outcomes included the presence of adverse events, hypoglycemia, and abnormalities in laboratory tests. All SAEs were resolved and none of the adverse events were considered by the investigators to be related to the study treatment. One child who was randomized but did not receive study treatment developed necrotizing enterocolitis. Hypoglycemia was not observed in any child, and blood glucose levels between the insulin and placebo groups were comparable. All patients, regardless of treatment group, who had data at discharge and at month three, presented insulin autoantibodies lower than 7 μU/mL and islet antigen-2 values lower than 0.75 μU/mL (no infant developed antibodies against insulin).

In relation to the risk of bias, Mank et al.^17^ presented some concerns, whereas Shehadeh et al.^18^ reported a low risk.

## DISCUSSION

During embryonic development, anatomically, the GIT after 20 weeks of gestation already resembles that of a full-term newborn. However, the functional development of premature infants is quite limited, with digestion and absorptive capacities being very restricted before 28 to 32 weeks, in addition to prolonged gastric emptying times.^19^

Due to GIT immaturity in premature neonates, various complications have been related to feeding intolerance, such as emetic episodes and abdominal distension. Furthermore, these patients are known to be at higher risk of sepsis and intestinal failure related to parenteral nutrition. Thus, the early introduction of minimal or trophic enteral nutrition has been encouraged, aiming to overcome the problem, often not with the objective of nutrition but rather to stimulate the maturation of GIT motility and reinforce the regulation of nervous and hormonal functions of motor activity.^6,9,20,21^

Although only two articles were eligible for this systematic review, the favorable findings presented a new perspective on mortality and complications prevention in the premature population. Both studies, Mank et al. and Shehadeh et al., obtained positive results regarding the time required to achieve full enteral nutrition, thereby reducing the exposure time of this population to the risks of parenteral nutrition.^17,18^

When analyzing the secondary outcomes, it is suggested that there is a decrease in SAEs in neonates who received the intervention. By reducing the occurrence of SAEs as well as the duration of exposure to enteral nutrition, enteral insulin is suggested to emerge as a promising measure to reduce morbidity, mortality, and healthcare system costs. Insulin plays an important role in intestinal maturation, being present in amniotic fluid (up to 20 μU/mL), and from 26 weeks of gestation, it becomes the primary source of insulin exposure in the GIT, contributing to development. Premature birth interrupts these fetal life processes.^19^

Mank et al. suggested that high doses of rh insulin (2000 μIU/mL of milk) should be the preferred for routine administration in clinical practice. However, they indicated that further studies are necessary before incorporating this practice into enteral nutrition guidelines for preterm infants. Additionally, they pointed out that long-term benefits, such as the effect on neurological development, deserve investigation. Shehadeh et al. studied only neonates who were using infant formula and emphasized that this allowed the exclusion of extremely premature neonates from the study, as many of them were more likely to be fed with breast milk.^17,18^

Among the limitations of this review, it is necessary to highlight the limited number of studies on the subject in question. Although both studies, Mank et al. and Shehadeh et al., are of adequate quality and have relevant outcomes, additional studies are needed to support the findings.^17,18^

Even though the study by Mank et al.^7^ is criticized by some authors, the amount of loss was controlled, and the study interruption criterion did not modify the similarity between the groups and did not result in bias.^1,7,16^

Additionally, as pointed out in both studies, further investigations that include extremely premature infants or those with extremely low birth weight are necessary, as this population presents higher morbidity related to prolonged parenteral and enteral nutrition. Thus, we suggest conducting studies with high methodological rigor, the inclusion of extremely premature infants or those with extremely low birth weight, statistical analysis related to adverse events, and determining the most appropriate dose of enteral insulin, as there is no defined consensus.^17,18^

In conclusion, administering enteral insulin in preterm infants was associated with a shorter time to achieve FEF. No statistical significance was observed for days of parenteral nutrition, mortality, and weight gain. Despite the relevant positive effects among the various outcomes analyzed, the evidence on the use of enteral insulin in premature infants is insufficient and should be further explored to recommend or not its use in clinical practice.

## Data Availability

The database that originated the article is available with the corresponding author.
